# Recent Advances in the Treatment of Breast Cancer

**DOI:** 10.3389/fonc.2018.00227

**Published:** 2018-06-14

**Authors:** Christy W. S. Tong, Mingxia Wu, William C. S. Cho, Kenneth K. W. To

**Affiliations:** ^1^Faculty of Medicine, School of Pharmacy, The Chinese University of Hong Kong, Hong Kong, Hong Kong; ^2^Department of Clinical Oncology, Queen Elizabeth Hospital, Hong Kong, Hong Kong

**Keywords:** breast cancer, cyclin-dependent kinases 4 and 6 inhibitors, hormone receptor, human epidermal growth factor receptor 2, poly(ADP-ribose) polymerase inhibitor, programmed cell death protein 1, trastuzumab, triple negative breast cancer

## Abstract

Breast cancer (BC) is the most common malignancy in women. It is classified into a few major molecular subtypes according to hormone and growth factor receptor expression. Over the past few years, substantial advances have been made in the discovery of new drugs for treating BC. Improved understanding of the biologic heterogeneity of BC has allowed the development of more effective and individualized approach to treatment. In this review, we provide an update about the current treatment strategy and discuss the various emerging novel therapies for the major molecular subtypes of BC. A brief account of the clinical development of inhibitors of poly(ADP-ribose) polymerase, cyclin-dependent kinases 4 and 6, phosphatidylinositol 3-kinase/protein kinase B/mammalian target of rapamycin pathway, histone deacetylation, multi-targeting tyrosine kinases, and immune checkpoints for personalized treatment of BC is included. However, no targeted drug has been approved for the most aggressive subtype—triple negative breast cancer (TNBC). Thus, we discuss the heterogeneity of TNBC and how molecular subtyping of TNBC may help drug discovery for this deadly disease. The emergence of drug resistance also poses threat to the successful development of targeted therapy in various molecular subtypes of BC. New clinical trials should incorporate advanced methods to identify changes induced by drug treatment, which may be associated with the upregulation of compensatory signaling pathways in drug resistant cancer cells.

## Introduction

Breast cancer (BC) is the most commonly diagnosed and the second leading cause of cancer-related deaths among women worldwide (1). One of the major challenges for its treatment is its heterogeneous nature, which determines the therapeutic options (2). By evaluating a few biomarkers, including the presence of hormone receptors (HRs), excess levels of human epidermal growth factor receptor 2 (HER2) protein, and/or extra copies of the *HER2* gene (3, 4), BC is classified into four major molecular subtypes: (i) luminal A (HR+/HER2−); (ii) HER2+; (iii) luminal B (HR+/HER2+); and (iv) triple negative (TNBC; HR−/HER2−; also overlap with the basal-like subtype). Each of these subtypes has different risk factors for incidence, therapeutic response, disease progression, and preferential organ sites of metastases.

Luminal BC is positive for HR [estrogen receptor (ER) and progesterone receptor (PR)]. It is subdivided into two subgroups (A and B). Luminal A subgroup (HR+/HER2−) is usually slow-growing and less aggressive than other subtypes. They are more responsive to hormonal interventions (5). Luminal B subgroup (HR+/HER2+) is further defined by its high expression of Ki67 (a proliferation marker) or HER2. Luminal B usually has a poorer prognosis than luminal A (5). HER2+ BC has overexpression or amplification of the *HER2*/*ERBB2* oncogene and may be treated with anti-HER2 therapies. Basal-like BC lacks HR and HER2, so they are also known as triple negative breast cancer (TNBC). Most BC patients (84%) have HR+ diseases, which includes 71% from HR+/HER− (luminal A) and 12% from HR+/HER2+ (luminal B). Only 5% of BC patients are HER2+ but HR−. TNBC makes up the remaining 12% of the total patient population (6).

## Current Treatment Regimens and Novel Therapies for Different Subtypes of BC

### Luminal BC (HR+ BC)

#### Current Treatment Regimens

Luminal BC, which is also hormone receptor positive (HR+), represents the vast majority (60–80%) of BC cases in developed countries (6) and this patient population is increasing in premenopausal women (7, 8).

For HR+ BC, endocrine therapy is the mainstay for treatment, which works by blocking the effects of hormone or lowering the hormone level. Currently available drugs include (i) tamoxifen, a prodrug that blocks estrogen uptake by the ER; (ii) aromatase inhibitors (letrozole, anastrozole, and exemestane), which suppress the conversion of androgens to estrogens, thus resulting in estrogen depletion; (iii) luteinizing hormone-releasing hormone analogs (goserelin and leuprolide), which suppress the production of hormone from the ovary; and (iv) fulvestrant (a selective ER degrader), which is suitable for BC patients refractory to previous hormonal therapy. Sequential administration of endocrine treatments are recommended until there is a need for rapid response or evidence of clinical resistance, when chemotherapy will be indicated (9).

Since endocrine drugs work by different mechanisms, they are generally used in combination for better anticancer efficacy. However, conflicting results have been reported (10–12). It is generally believed that patients with endocrine therapy-naïve advanced BC and those with highly endocrine-sensitive tumors may benefit the most from combination endocrine therapy (13).

#### Novel Therapies

Metastatic HR+ BC may develop resistance to standard hormonal therapies, which was mediated by genomic alterations in the ER and/or upregulation of other signaling pathways. Therefore, the development of new agents has aimed at reversing resistance to hormonal therapies (Table 1).

**Table 1:** Novel drugs for treating different molecular subtypes of breast cancer (BC).

Drug (alternative names)	Mode of action	Targeted population	Monotherapy or combination therapy	Latest stage of clinical development
**I. For treating HR+ BC**				

Palbociclib (Ibrance^®^)	Oral small-molecule inhibitor of cyclin-dependent kinase CDK4 and CDK6	Advanced stage, HER2−	Combination therapy with letrozole	Approved by US FDA (February 2015)

		Advanced stage, pretreated, HER2−	Combination therapy with fulvestrant	Phase III

Ribociclib (Kisqali^®^)	Oral small-molecule inhibitor of CDK4 and CDK6	Advanced stage, HER2−	Combination therapy with letrozole	Approved by US FDA (March 2017)

		Advanced stage, pretreated, HER2−	Combination therapy with fulvestrant	Phase III (ongoing)

Abemaciclib (LY2835219)	Oral small-molecule inhibitor of CDK4 and CDK6	Advanced stage, HER2−	Combination therapy with letrozole	Phase III (ongoing)

		Advanced stage, pretreated, HER2−	Combination therapy with fulvestrant	Phase III

Buparlisb (BKM120)	Oral small-molecule inhibitor of pan-class I phosphatidylinositol 3-kinase (PI3K)	Advanced stage, pretreated, HER2−	Combination therapy with fulvestrant	Phase III

		Early stage, HER2−	Combination therapy with letrozole	Phase II (ongoing)

Pictilisib (GDC-0941)	Oral small-molecule inhibitor of pan-class I PI3K	Advanced stage, pretreated, HER2−	Combination therapy with fulvestrant	Phase II (will not be further pursued)

		Early stage, HER2−	Combination therapy with anaestrozole	Phase II

Pilaralisib (SAR245408)	Oral small-molecule inhibitor of pan-class I PI3K	Advanced stage, pretreated, HER2−	Combination therapy with letrozole	Phase I/II (will not be further pursued)

Voxtalisib (SAR245409)	Oral small-molecule inhibitor of pan-class I PI3K and mammalian target of rapamycin (mTOR)	Advanced stage, pretreated, HER2−	Combination therapy with letrozole	Phase I/II (will not be further pursued)

Alpeisib (BYL719)	Oral small-molecule inhibitor of α-specific class I PI3K	Advanced stage, pretreated, HER2−	Combination therapy with fulvestrant	Phase III (ongoing)

		Early stage, HER2−	Combination therapy with letrozole	Phase II (ongoing)

Taselisib (GDC-0032)	Oral small-molecule inhibitor of α-specific class I PI3K	Advanced stage, pretreated, HER2−	Combination therapy with fulvestrant	Phase II (ongoing)

Everolimus (Afintor^®^)	Oral small-molecule inhibitor of mTOR	Advanced stage, pretreated	Combination therapy with exemestane	Approved by US FDA (July 2012)

Temsirolimus (Torisel^®^)	Oral small-molecule inhibitor of mTOR	Advanced stage	Combination therapy with letrozole	Phase III

		Advanced stage, pretreated	Monotherapy	Phase II (will not be further pursued)

Entinostat	Histone deacetylase (HDAC) inhibitor	Advanced stage, pretreated	Combination therapy with exemestane	Phase III (ongoing)

Vorinostat	HDAC inhibitor	Advanced stage, pretreated	Combination therapy with tamoxifen	Phase II

**II. For treating HER2+ BC**				

Buparlisb (BKM120)	Oral small-molecule inhibitor of pan-class I PI3K	Advanced stage, pretreated	Combination therapy with lapatinib	Phase Ib

		Advanced stage, pretreated	Combination therapy with trastuzumab and paclitaxel	Phase II

Pilaralisib (SAR245408)	Oral small-molecule inhibitor of pan-class I PI3K	Advanced stage, pretreated	Combination therapy with trastuzumab/trastuzumab and paclitaxel	Phase I/II

MK-2206	Oral small-molecule inhibitor of protein kinase B	Advanced stage, pretreated	Combination therapy with trastuzumab	Phase I

Everolimus (Afintor^®^)	Oral small-molecule inhibitor of mTOR	Advanced stage, pretreated	Combination therapy with trastuzumab and vinorelbine	Phase III

Ridaforolimus (MK-8669)	Oral small-molecule inhibitor of mTOR	Advanced stage, pretreated	Combination therapy with trastuzumab	Phase IIb

Sirolimus	Oral small-molecule inhibitor of mTOR	Advanced stage, pretreated	Combination therapy with trastuzumab	Phase II

Neratinib (HKI-272)	Irreversible binder of HER1, HER2, and HER4	Early stage, pretreated	Monotherapy	Phase III

Patritumab (AMG 888, U3-1287)	Anti-HER3 monoclonal antibody	Advanced stage	Combination therapy with trastuzumab and paclitaxel	Phase Ib

Margetuximab (MGAH22)	Anti-HER2 monoclonal antibody	Advanced stage	Monotherapy	Phase I

Lonafarnib (SCH66336)	Farnesyl transferase inhibitor	Advanced stage	Combination therapy with trastuzumab and paclitaxel	Phase I

Nelipepimut-S (E75)	Therapeutic peptide vaccine	Early stage	Combination therapy with trastuzumab	Phase II (ongoing)

Recombinant HER2 protein (dHER2)	Therapeutic peptide vaccine	Early stage	Monotherapy	Phase I

		Advanced stage	Monotherapy	Phase I/II

		Advanced stage, pretreated	Combination therapy with lapatinib	Phase I

**III. For treating triple negative breast cancer**				

Olaparib (Lynparza^®^)	Oral PARP inhibitor	Advanced stage, HER2−, gBRCA+	Monotherapy	Phase III

Talazoparib (BMN 673)	Oral PARP inhibitor	Advanced stage, HER2−, gBRCA+	Monotherapy	Phase III (ongoing)

Veliparib (ABT-888)	Oral PARP inhibitor	Advanced stage, HER2−, gBRCA+	Combination therapy with carboplatin and paclitaxel	Phase III (ongoing)

Niraparib (Zejula^®^)	Oral PARP inhibitor	Advanced stage, HER2−, gBRCA+	Monotherapy	Phase III (ongoing)

			Combination therapy with pembrolizumab	Phase I/II (ongoing)

Rucaparib (Rubraca^®^)	Oral PARP inhibitor	Advanced stage, HER2−, gBRCA+	Monotherapy	Phase II (ongoing)

			Combination therapy with cisplatin	Phase II (ongoing)

Glembatumumab vedotin	Antibody-drug conjugate	Advanced stage, pretreated, gpNMB+	Monotherapy	Phase II (ongoing)

Bicalutamide (Casodex^®^)	Androgen-receptor inhibitor	Advanced stage, AR+, HR−	Monotherapy	Phase II

Pembrolizumab (Keytruda^®^)	Anti-PD-1 monoclonal antibody	Advanced stage	Monotherapy	Phase II (ongoing)

##### Cyclin-Dependent Kinases 4 and 6 (CDK4/6) Inhibitors

Among the emerging therapies, CDK4/6 inhibitors (palbociclib, ribociclib, and abemaciclib) have attracted the most attention. CDK4/6 regulate cell cycle progression by their reversible interaction with cyclin D1. Approximately 29 and 14% of patients with HR+/HER2− BC were found to have amplification of cyclin D1 and CDK4, respectively. Importantly, even when hormonal resistance developed, the tumors still depend on CDK4/6-cyclin D1 for proliferation (14). Therefore, more pronounced G1-S cell cycle arrest was observed in HR+/HER2− BC after treatment with combination of hormonal therapy and CDK4/6 inhibitor (15). CDK4/6 inhibitors work by blocking the phosphorylation of retinoblastoma protein, thereby downregulating E2F-response genes to mediate G1-S arrest. They also dephosphorylate the transcription factor Forkhead box protein M1 to inhibit cell proliferation (15).

Palbociclib and ribociclib have received FDA approval for combination with aromatase inhibitor as first-line treatment of HR+/HER2− advanced BC. They were shown to significantly improve median PFS by 10 months (16) and PFS rate by 20% after 18 months (17), respectively, compared to letrozole alone. On the other hand, abemaciclib is still under phase III investigation (NCT02246621). As second-line treatment in combination with fulvestrant in HR+/HER2− advanced BC, palbociclib and abemaciclib were demonstrated to significantly prolong median PFS by 5 months (18) and 7 months (19), respectively, compared to fulvestrant alone. Ribociclib is in phase III investigation (NCT02422615). Although all three CDK4/6 inhibitors worked through similar mechanism, abemaciclib exhibited a higher monotherapy response rate and induced less neutropenia, which may be related to its superior CDK4 inhibition (20).

##### Inhibitors Targeting Phosphatidylinositol 3-Kinase (PI3K)/Protein Kinase B (Akt)/Mammalian Target of Rapamycin (mTOR) Pathway

Aberrant activation of the PI3K–Akt–mTOR signaling pathway is known to contribute to hormonal resistance (21). This pathway is activated in over 70% BC, with the PI3K catalytic subunit p110α (*PIK3CA*) being one of the most frequently mutated and/or amplified genes (22). Combination therapies targeting both HR and PI3K/Akt/mTOR pathways have been evaluated to reverse hormone resistance (21).

###### PI3K Inhibitors

The combination of PI3K inhibitors with aromatase inhibitor has been used as second-line treatment for HR+/HER− advanced BC. While buparlisib (a pan-class I PI3K inhibitor) has been shown to significantly improve PFS, especially in those who also have *PIK3CA* mutation, buparlisib (23), pictillisib (24), pilaralisib (25), and voxtalisib (also an mTOR inhibitor) (25) did not give rise to significant clinical benefit due to high toxicities. The more selective and less toxic α-specific PI3K inhibitors (alpeisib and taselisib), currently in phase III trials (NCT02437318 and NCT02340221), were found to exhibit promising efficacy, particularly in BC patients who had *PIK3CA* mutation (26, 27).

As neoadjuvant treatment in combination with letrozole or anastrozole for HR+/HER2− early BC, both pictillisib (28) and taselisib (29) were found to enhance antitumor effects irrespective of *PIK3CA* status. Buparlisb and alpelisib are under phase II investigation (NCT01923168).

###### mTOR Inhibitors

Everolimus has received US FDA approval for HR+ advanced BC in combination with exemestane after treatment failure with letrozole or anastrozole (30). However, temsirolimus failed to show any clinical benefits either as first-line treatment in combination with letrozole (31) or as second-line therapy as a single agent (32) in advanced HR+ BC.

###### Histone Deacetylase (HDAC) Inhibitors

Hormonal resistance is also caused by histone deacetylation-mediated loss of ER expression in ER+ patients (33). This may be reversed by HDAC inhibitors, which upregulated expression of ERα and aromatase and inhibited growth factor signaling pathways (34). As second-line treatment for HR+ advanced BC [phase III (NCT02115282)], both entinostat and vorinostat exhibited superior anticancer activity in combination with exemestane (35) and tamoxifen (36) respectively, compared to exemestane/tamoxifen alone.

###### Steroid Sulfatase Inhibitors

Steroid sulfatase is a key enzyme regulating the conversion of inactive sulfate-conjugated steroids to active and estrogenic non-conjugated forms (37). The expression level and enzyme activity of steroid sulfatase were found to be remarkably increased in ERα-positive BC (38). Thus, inhibition of steroid sulfatase represents a logical approach for reducing estrogenic steroids that may stimulate BC growth. A recent phase II trial showed that combination of irosustat (first-generation steroid sulfatase inhibitor) and aromatase inhibitor was well-tolerated and resulted in clinical benefit (39). Another novel dual-acting steroid sulfatase inhibitor (SR16157), which directly inhibits steroid sulfatase and releases a selective ERα modulator, has been evaluated in hormone-dependent BC (40).

### HER2+ BC

#### Current Treatment Regimens

For HER2+ BC, several molecular targeted agents have been approved to be used alone or in combination with standard chemotherapy. They include (i) trastuzumab (anti-HER2 monoclonal antibody); (ii) pertuzumab (anti-HER2 monoclonal antibody with a different binding site on HER2 than trastuzumab); (iii) ado-trastuzumab emtansine, an antibody-cytotoxic agent conjugate consisting of trastuzmab linked with a small-molecule microtubule inhibitor (emtansine); and (iv) lapatinib, a dual tyrosine kinase inhibitor (TKI) that interrupts both HER2 and epidermal growth factor receptor (EGFR) pathways. BC patients are tested for *HER2* gene amplification or protein overexpression to determine whether they would benefit from anti-HER2 therapy.

In early-stage HER2-positive BC, neoadjuvant treatment with a combination of chemotherapy and anti-HER2 targeted therapy is currently the standard regimen (41). This is followed by surgery, radiotherapy, and another 12-month HER2-targeted therapy. Endocrine adjuvant therapy may also be added depending on specific cancer biology. With the introduction of HER2-targeted therapies over the past 15 years, the median overall survival (OS) of patients with HER2+ advanced BC has increased substantially from approximately 20 months to currently about 5 years (42, 43).

#### Novel Therapies

The emergence of primary and acquired resistance to trastuzumab is severely limiting its clinical utility in HER2+ BC. Elucidation of the resistance mechanisms and discovery of targeted agents and immunotherapies have resulted in improved treatment outcomes (Table 1).

##### PI3K/Akt/mTOR Inhibitors

Combinations of PI3K/Akt/mTOR inhibitors with trastuzumab have been studied to overcome trastuzumab resistance mediated by aberrant activation of the pathway. Pan-class I PI3K inhibitors (buparlisib and pilaralisib), when combined with lapatinib (44), trastuzumab (45), or trastuzumab and paclitaxel (46), were found to demonstrate promising efficacy and safety in patients with pretreated HER2+ advanced BC. Akt inhibitor (MK-2206) was found to exhibit favorable antitumor activities when combined with trastuzumab (47) or trastuzumab and paclitaxel (48) in pretreated patients with HER2+ advanced BC. As for the mTOR inhibitor, the combination of everolimus (an mTOR inhibitor) with trastuzumab and vinorelbine did not significantly improve clinical outcome in pretreated HER+ BC patients (49). However, the combination was found to produce better anticancer activity than trastuzumab alone in HER+ patients who are also HR− (49). On the other hand, the combination of two newer mTOR inhibitors, ridaforolimus (50) and sirolimus (51), with trastuzumab have also shown promising activity in refractory HER2+ BC.

##### Inhibitors Targeting HER-Family Receptors

Growth factor ligands of HER-family receptors [HER1 (EGFR), HER3, or HER4] are known to inhibit the anticancer effect of trastuzumab (52). Moreover, overexpression of HER2/HER3 heterodimers, which are more active than other heterodimers or homodimers formed by HER family (53), have been reported to cause trastuzumab resistance. Therefore, a broader inhibition of HER-family receptors may elicit greater anticancer effect than trastuzumab alone.

###### Multi-Targeting TKIs

Neratinib, an irreversible TKI of HER1/HER2/HER4, has been reported to significantly improve the 2-year invasive disease-free survival after trastuzumab-based adjuvant therapy in HER2+ BC (54).

###### Monoclonal Antibodies

Patritumab (anti-HER3 monoclonal antibody) showed promising antitumor activity in preclinical study through inhibiting the formation of HER2/HER3 heterodimers. It was found to exhibit favorable efficacy and acceptable tolerability in patients with HER2+ advanced BC (55). Margetuximab (targeting HER2) was well-tolerated and it demonstrated promising single-agent activity in a first-in-man phase I trial in HER2+ advanced BC (56). Further clinical trials are ongoing to investigate its usefulness as a single agent (NCT02492711) or in combination with pembrolizumab (NCT02689284) (56).

###### Antibody-Drug Conjugate (ADC)

Trastuzumab emtansine is an ADC that incorporates the HER2-targeting activity of trastuzumab with the cytotoxicity of a microtubule-inhibitory drug (57). It is approved for second-line treatment in trastuzumab/lapatinib-relapsed/refractory HER2+ BC (58, 59).

###### Farnesyl Transferase Inhibitors (FTI)

Lonafarnib, as a specific FTI, inhibits Ras function by farnesylation. Although *RAS* mutations are not common (<2%) in BC, Ras protein and its downstream effectors are often activated due to overexpression of upstream signaling molecules (e.g., HER2) (60). Recently, a phase I trial showed that the addition of lonafarnib to trastuzumab and paclitaxel therapy exhibited superior antitumor activities in HER2+ advanced BC (61).

###### Immunotherapy

Nelipepimut-S is a short peptide (HER2/neu 369–377, KIFGSLAFL) from the extracellular domain of HER2 (62). It was investigated as a vaccine to prevent clinical recurrence in high-risk BC patients (63). The combination use of nelipepimut-S and trastuzumab in HER2+ early BC is now studied in a phase IIb trial (NCT02297698). Another protein vaccine, recombinant HER2 protein (dHER2) was also found to exhibit immunogenicity to induce T-cell-mediated cytotoxicity in HER2+ early BC patients as an adjuvant treatment (64), in HER2+ advanced BC patients (65) as a single agent and in HER2+ advanced BC patients refractory to trastuzumab+ lapatinib (66).

### Triple Negative Breast Cancer

#### Current Treatment Regimens

Triple negative breast cancer is more aggressive and difficult to treat than HR+ and HER2+ BC. For TNBC, standard chemotherapy remains the mainstay of treatment. Interestingly, TNBC is the BC subtype with the most complete response to chemotherapy (22%). However, their recurrence and metastasis rates are higher than those carrying non-TNBC tumors (67). The median OS for patients with metastatic TNBC is about 9–12 months with conventional cytotoxic agents. The lack of ER, PR, and HER2 expression precludes the use of targeted therapies in advanced TNBC, and the only approved systemic treatment option is chemotherapy [usually taxanes, anthracycline, and platinum drugs (68)] with or without bevacizumab [a recombinant humanized monoclonal antibody against vascular endothelial growth factor (VEGF)]. Given the suboptimal treatment outcome with chemotherapy, new targeted therapies for TNBC are badly needed.

#### Novel Therapies

**Figure 1:**
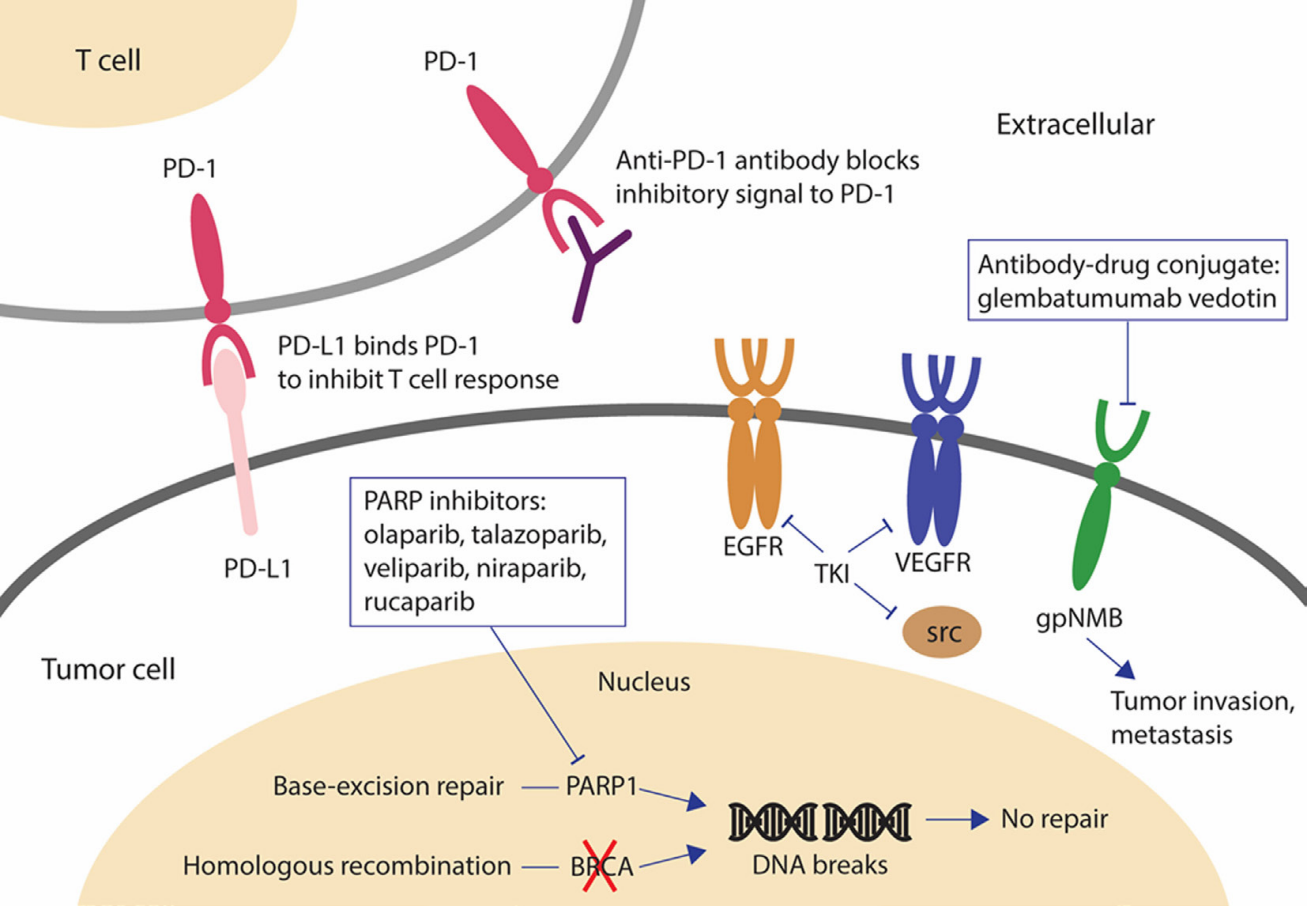
Novel drugs under investigation for triple negative breast cancer (TNBC). PARP inhibitors are effective in *BRCA*-mutated breast cancer (BC). When BRCA function is absent and PARP is inhibited, cancer cells are unable to repair DNA damage by homologous recombination or base-excision repair and cell death results. The antibody-drug conjugate, glembatumumab vedotin, may be effective in gpNMB-overexpressing BC by releasing the cytotoxic drug into gpNMB-expressing tumor cells, resulting in a targeted-cell killing effect. Tyrosine kinase inhibitors against EGFR, VEGFR, and SRC have been investigated for the treatment of TNBC because these signaling receptors mediating cancer cell growth are overexpressed or dysregulated in TNBC. The monoclonal antibody, pembrolizumab, may be effective regardless of PD-L1 expression by inducing an immune response to kill cancer cells. Abbreviations: PARP, poly(ADP-ribose) polymerase; gpNMB, glycoprotein NMB; AR, androgen receptor; DHT, dihydrotestosterone; PD-1, programmed cell death 1 receptor; PD-L1, ligand of programmed cell death 1 receptor; EGFR, epidermal growth factor receptor.

Among all BC subtypes, TNBC has the fewest therapeutic options due to the lack of well-defined molecular target(s). Identification of new therapeutic targets and development of effective targeted agents is urgently needed. Table 1 and Figure 1 summarize the promising agents currently in clinical development for TNBC.

##### Poly(ADP-ribose) Polymerase (PARP) Inhibitors

The most important advancement toward understanding the complex heterogeneity of TNBC is probably the discovery of a subgroup of sporadic TNBC that shares the homologous repair deficiency characteristic with BRCA1/2-mutated BC. Drug combination regimens are thus proposed by incorporating PARP inhibtors or the DNA-targeting platinum drug (carboplatin) (69, 70) to standard chemotherapy (71, 72).

The PARP enzyme repairs DNA single-strand breaks whereas the *BRCA1/BRCA2* genes encode tumor-suppressor proteins that repair DNA double-strand breaks through homologous recombination. PARP inhibitors have showed promising clinical activities in patients bearing germline *BRCA1/BRCA2* mutation (gBRCA+), presumably by synthetic lethality from unresolved DNA damage and by replication arrest caused by physical obstruction of DNA replication forks (73).

Olaparib has proceeded the furthest in clinical development. In a phase III trial, it improved median PFS by 2.8 months and lowered the risk of disease progression/death by 42% compared to standard chemotherapy (71). Talazoparib, currently in phase III trial (NCT01945775), has the greatest preclinical potency due to its strong binding to DNA by trapping PARP–DNA complexes (74). It demonstrated encouraging antitumor activities as a single agent in advanced gBRCA+ BC (75). Veliparib combined with carboplatin and paclitaxel, though failed to prolong PFS in gBRCA+ BC (76), is being investigated in phase III trial (NCT02163694) in advanced gBRCA+ BC (77). Niraparib (phase III, NCT01905592) and rucaparib (phase II, NCT02505048) are being investigated in gBRCA+ advanced BC patients as monotherapy and also in combination with chemotherapy (niraparib: phase I/II, NCT02657889; rucaparib: phase II, NCT01074970).

The use of PARP inhibitors or carboplatin in TNBC is usually determined by three DNA-based homologous recombination deficiency scores, which are highly correlated with genetic defects in *BRCA1/2* (78). However, none of these agents is effective in treating all TNBC because TNBC can be further divided into at least six subclasses [basal-like (BL1 and BL2), an immunomodulatory, a mesenchymal, a mesenchymal stem-like, and a luminal androgen receptor subtype], each of which has its own molecular features and unique drug sensitivity (79–81). The identification and characterization of clinically relevant molecular biomarkers of drug responsiveness is needed to further refine this treatment strategy.

##### Anti-Angiogenic Agents

The intra-tumoral expression of VEGF, a key angiogenic factor, is known to be remarkably higher in TNBC than in non-TNBC BC (82). Bevacizumab (anti-VEGF monoclonal antibody) suppresses tumor neovasculature growth and inhibits metastasis. In metastatic TNBC (phase III), the addition of bevacizumab to first-line chemotherapy (docetaxel) has been shown to increase response rate (placebo plus docetaxel: 46% versus bevacizumab plus docetaxel: 64%) and median PFS (placebo plus docetaxel: 8.1 months versus bevacizumab plus docetaxel: 10.0 months) (HR, 0.67; *P* < 0.001) (83, 84). Importantly, combination of bevacizumab with docetaxel did not affect significantly the overall safety profile of the regimen.

##### EGFR Inhibitors

Epidermal growth factor receptor is overexpressed in TNBC. Numerous phase II studies have recently evaluated the efficacy of cetuximab (anti-EGFR monoclonal antibody) in combination with cisplatin in metastatic TNBC (85, 86). While only modest objective response rate (ORR) was observed (ORR = 20% for cisplatin plus cetuximab versus 10% for cisplatin alone), cisplatin plus cetuximab resulted in longer median PFS (3.7 versus 1.5 months) and median OS (12.9 versus 9.4 months) compared with cisplatin alone. Current effort is being made to identify a sub-population of TNBC patients that may be more likely to respond to EGFR inhibitors (87). Favorable response may be correlated with lower expression of alpha-crystallin B chain, higher expression of PTEN, and lack of KRAS expression in the tumors (87).

##### SRC Inhibitors

SRC is a non-receptor signaling kinase downstream of several growth factor receptors (EGFR, IGF-1R, PDGFR, and HGFR), which is/are deregulated in TNBC. Dasatinib (inhibitor of multiple tyrosine kinases including SRC), when tested as a single agent for TNBC in a prospective, open label, phase II trial (CA180059), has shown disappointing result (88). Objective response rate (ORR) was only 4.7%. Median PFS was 8.3 weeks. Higher dose (100 mg BID) was associated with treatment interruption, dose reduction, and serious adverse events (88). However, in cell line studies, when dasatinib was combined with cetuximab (anti-EGFR monoclonal antibody) and cisplatin, synergistic anticancer activity in a panel of TNBC cell lines was observed (89). The three-drug combination produced more pronounced induction of apoptosis and inhibition of EGFR and MAPK phosphorylation than either single or two-drug combination (89). Moreover, cancer cell migration and invasion was also significantly inhibited by dasatinib alone treatment or dasatinib-containing combination treatment in TNBC cell lines (89). Therefore, clinical studies may be warranted to investigate the use of dasatinib-containing combinations in TNBC patients whose tumors co-overexpressed both EGFR and c-Src.

##### Monoclonal Antibodies

Glembatumumab vedotin is a monoclonal antibody-cytotoxic drug conjugate designed to target glycoprotein NMB-overexpressing (gpNMB+) TNBC (90). gpNMB is a transmembrane protein associated with tumor invasion and metastasis and it is overexpressed in 40% of TNBC (91). On gpNMB+ advanced TNBC patients (phase II trial), a significantly improved PFS and OS by glembatumumab vedotin was observed compared to the treatment of physician’s choice (92).

##### Immunotherapies

Pembrolizumab is a human monoclonal IgG4-ĸ antibody against the programmed cell death 1 receptor (PD-1). It demonstrated clinical efficacy and safety in patients with advanced TNBC. PD-1 prevents autoimmunity by suppressing T cells and thus preventing the immune system from killing cancer cells. While patients with PD-L1 (a ligand of PD-1)-positive advanced TNBC were selected for investigation in a phase Ib study (93), the antitumor activity of pembrolizumab appeared to be independent of PD-L1 expression according to another ongoing phase II study (94). Importantly, pembrolizumab also showed durable antitumor activity in patients with heavily pretreated metastatic TNBC (94).

## Conclusion

With the advancements in the chemotherapy for BC, the mortality rate from BC is decreasing in the last decade. Targeting ER has proved one of the most powerful treatment modalities against HR+ BC (95). Moreover, the success of the biological drugs such as anti-HER2 monoclonal antibody (96) also highlighted the feasibility and significance of the molecular targeting approach in BC therapy. However, metastasizing TNBC remains a deadly disease with limited treatment options. In recent years, the molecular mechanisms driving the heterogeneous treatment response in BC are better elucidated. This has fueled the development of novel targeted agents, including inhibitors of PARP, CDK4/6, PI3K/AKT/mTOR, multiple kinases, or immune checkpoint, for the treatment of specific molecular subtypes of BC. Treatment options should be tailored to individual patient accordingly.

## Author Contributions

CT, MW, WC, and KT: design, collection of data, manuscript, editing, approval of final version, and accountability.

## Conflict of Interest Statement

The authors declare that the manuscript was prepared in the absence of any commercial or financial relationships that could be construed as a potential conflict of interest.

## Abbreviations

ADC, antibody-drug conjugate; Akt, protein kinase B; AR, androgen receptor; BC, breast cancer; CDK4/6, cyclin-dependent kinases 4 and 6; EGFR, epidermal growth factor receptor; ER, estrogen receptor; FOXM1, Forkhead box protein M1; HDAC, histone deacetylase; HR, hormone receptor; HER2, human epidermal growth factor receptor 2; mTOR, mammalian target of rapamycin; PARP, poly(ADP-ribose) polymerase; PD-1, programmed cell death 1 receptor; PD-L1, ligand of programmed cell death 1 receptor; PI3K, phosphatidylinositol 3-kinase; PIK3CA, PI3K catalytic subunit p110α; PR, progesterone receptor; Rb, retinoblastoma protein; TNBC, triple negative breast cancer; VEGF, vascular endothelial growth factor.
